# Profile of the GSK Published Protein Kinase Inhibitor Set Across ATP-Dependent and-Independent Luciferases: Implications for Reporter-Gene Assays

**DOI:** 10.1371/journal.pone.0057888

**Published:** 2013-03-07

**Authors:** Patricia Dranchak, Ryan MacArthur, Rajarshi Guha, William J. Zuercher, David H. Drewry, Douglas S. Auld, James Inglese

**Affiliations:** 1 National Center for Advancing Translational Sciences, National Institutes of Health, Rockville, Maryland, United States of America; 2 National Human Genome Research Institute, National Institutes of Health, Bethesda, Maryland, United States of America; 3 Department of Chemical Biology, GlaxoSmithKline, Research Triangle Park, North Carolina, United States of America; Albert-Ludwigs-University, Germany

## Abstract

A library of 367 protein kinase inhibitors, the GSK Published Kinase Inhibitor Set (PKIS), which has been annotated for protein kinase family activity and is available for public screening efforts, was assayed against the commonly used luciferase reporter enzymes from the firefly, *Photinus pyralis* (FLuc) and marine sea pansy, *Renilla reniformis* (RLuc). A total of 22 compounds (∼6% of the library) were found to inhibit FLuc with 10 compounds showing potencies ≤1 µM. Only two compounds were found to inhibit RLuc, and these showed relatively weak potency values (∼10 µM). An inhibitor series of the VEGFR2/TIE2 protein kinase family containing either an aryl oxazole or benzimidazole-urea core illustrate the different structure activity relationship profiles FLuc inhibitors can display for kinase inhibitor chemotypes. Several FLuc inhibitors were broadly active toward the tyrosine kinase and CDK families. These data should aid in interpreting the results derived from screens employing the GSK PKIS in cell-based assays using the FLuc reporter. The study also underscores the general need for strategies such as the use of orthogonal reporters to identify kinase or non-kinase mediated cellular responses.

## Introduction

A significant challenge in small molecule HTS is to effectively differentiate between compounds that demonstrate genuine activity against the biological target or pathway of interest from compounds that interfere with the assay format or method [1]. For reporters and sensors used in bioassay development, a profile of their inhibition by library compounds is useful in understanding the potential non-target mediated activities to which the assay may be susceptible. Luciferases are one of the most common reporter enzymes used to construct cell-based assays [2], [3]. The firefly luciferase derived from *Photinus pyralis* (FLuc) is the most widely used luciferase [4]. Another commonly used luciferase is derived from the sea pansy, *Renilla reniformis* (RLuc), and is unrelated to FLuc [5], which has enabled the construction of cell-based assays using a dual-luciferase strategy [6], [7]. Both FLuc and RLuc bind different low-molecular weight (LMW) luciferin substrates and FLuc requires ATP for production of bioluminescence [8], [9], [10]. Not unexpectedly, both enzymes can be inhibited by low molecular weight compounds (∼500 MW) found in typical compound libraries [11], [12] which can confound the interpretation of cell-based assays that employ these enzymes in high throughput screening (HTS) [3]. An insidious aspect of some luciferase inhibitors is that these can lead to increases in bioluminescence, mimicking gene/pathway activation, due to inhibitor-based enzyme stabilization [13], [14]. In fact it has been found that FLuc inhibitors show large enrichments in FLuc-based cellular assays, but not in assays using alternative detection methods, regardless if the aim of the assay was to identify agonists or antagonist of the bioluminescence response [11], [13].

Certain protein kinase inhibitors have been identified as luciferase inhibitors, such as the VEGF/EGRF tyrosine kinase inhibitor SU4312 against FLuc [15] and the PKA inhibitor H89 against RLuc [12]. This activity needs to be considered when interpreting results for these protein kinase inhibitors using cell-based assays that employ luciferases. Recently, GlaxoSmithKline (GSK) released a set of 367 ATP-competitive kinase inhibitors from published accounts of proprietary drug discovery efforts (PKIS: published kinase inhibitor set). PKIS includes compounds active at their original target kinase and importantly compounds inactive at their original kinase target. This range allows for the elucidation of structure activity relationships (SAR) at a particular kinase and also provides greater opportunity (via more structural diversity within a series) for interaction with new kinases. The set is accompanied with well-characterized activity annotation, including data from a panel of over 200 kinase assays. We were interested in annotating this list of compounds with FLuc and RLuc inhibitory activity because this information should help guide the use of these compounds in cell-based assays. We measured concentration-response inhibition for compounds in the GSK PKIS in assays using purified enzyme preparations of FLuc and RLuc and K_M_ levels of substrates. We noted that relatively few compounds inhibited RLuc and those that did had weak potency values (∼10 µM), however approximately 10% of the library inhibited FLuc with some inhibitors showing potencies <1 µM. These results are described here to help guide scientists employing this library in their research.

## Materials and Methods

### The GlaxoSmithKline Published Kinase Inhibitor Set (PKIS)

The PKIS is available in screening quantities from GSK, free of charge. GSK typically provides 10 µL of a 10 mM solution in DMSO to scientists who wish to test the compounds. The set can be dispensed in 96- or 384-well plates. In order to obtain the compounds, interested institutions must fill out and agree to a streamlined material transfer agreement (MTA). The primary stipulation in the MTA is that the screening results must be made publicly available so that all may benefit. Readers interested in obtaining the set should contact William Zuercher of GlaxoSmithKline at william.j.zuercher@gsk.com.

### FLuc and RLuc enzymatic assays

To determine compound potency against purified luciferase enzymes, 3 µL of luciferase substrate was dispensed to each well of 1536-well white/solid bottom plates (Greiner Bio-One North America) using the BioRAPTR FRD (Beckman Coulter), for a final concentration of 10 µM D-luciferin (Sigma) and 10 µM ATP or 5 µM coelenterazine-H (Promega, Madison, WI). 23 nL of compounds were transferred using a 1536-pin tool (Wako) into assay wells, resulting in a final concentration range of ∼0.3 nM to 57 µM with 12 titration points. DMSO and titrations of PTC124 positive control from a top concentration of 57 µM (S6003, Selleck Chemicals, Houston, TX) and BTS (Tocris Bioscience, Minneapolis, MN) positive control from a top concentration of 230 µM plated in rows 31–32 in each plate served as luciferase inhibitory controls. 1 µL of purified luciferase was then dispensed into each well for final concentrations 10 nM FLuc or 1 nM RLuc. The bioluminescence outputs were measured by an EnVision reader (PerkinElmer, Wellesley, MA). Due to the relatively short luminescence duration of the RLuc assay, dispense time constants were set to match closely to the read time of the plate reader. As a result, each individual well was read with approximately the same time delay from reagent dispense to signal acquisition. See **Table 1** for the 1536-well assay protocols.

**Table 1:** FLuc or RLuc assay (1536-well plate format).

Step	Parameter	Value	Description
1	Reagent	3 µL	luciferase substrates; white/solid bottom plates
2	Compounds	23 nL	Pin tool delivery
3	Reagent	1 µL	luciferase enzyme
4	Incubation time	5 or 2 min	room temperature, protected from light
5	Assay read	4 min	EnVision plate reader
*Notes*			
2	Delivered with Wako 1536 Pintool. Final compound concentration ranged from 0.32 nM to 57 µM		
3-FLuc	Plates from Greiner Bio-One North America; Final enzyme concentration was 10 nM *P. pyralis* (FLuc); final substrate concentration: 10 µM D-luciferin (Sigma) and 10 µM ATP; reagent delivery by BioRaptor FRD (Beckman Coulter)		
3-RLuc	Plates from Greiner Bio-One North America; Final enzyme concentration 1 nM *Renilla* luciferase (RLuc); final substrate concentration: 5 µM coelenterazine-H (Promega); reagent delivery by BioRaptor FRD (Beckman Coulter)		
4	5 min incubation was used for FLuc and 2 min for RLuc.		

### Data analysis for qHTS

Data from each assay was normalized plate-wise to corresponding intraplate controls as described previously [16], [17]. The same control data sets were also used for the calculation of Z′ factors from each assay. In all assays, wells containing DMSO were used as no-response controls. For the FLuc enzymatic assay, each compound-treated well on the plate was normalized to the average readings of n = 24 PTC124 treated wells and n = 48 DMSO wells from the same plate. Wells were scaled to a full range of DMSO and zero, and then normalized using the maximum response observed in the screen as 100% activity. For the RLuc enzymatic assay, n = 24 BTS treated wells for the positive control, n = 48 DMSO. MSR values were calculated using the cumulative potency differences of the de-interleaved duplicate control titrations from each plate as previously described [18]. The normalized data from each assay plate set was corrected using DMSO-only treated assay plates read at the start and end of each assay run, and the resulting inter-plate titration data fit, using in-house software, to the standard Hill equation and dose-response curves automatically classified as described previously [16].

### Database deposition

The data generated in this study has been deposited in PubChem, http://pubchem.ncbi.nlm.nih.gov. The FLuc AID is 652016 and RLuc AID is 652015. The FLuc data can also be found under the broader FLuc profiling summary AID 2309.

### Concentration response curve fitting and curve classification assignments

**Figure 1:**
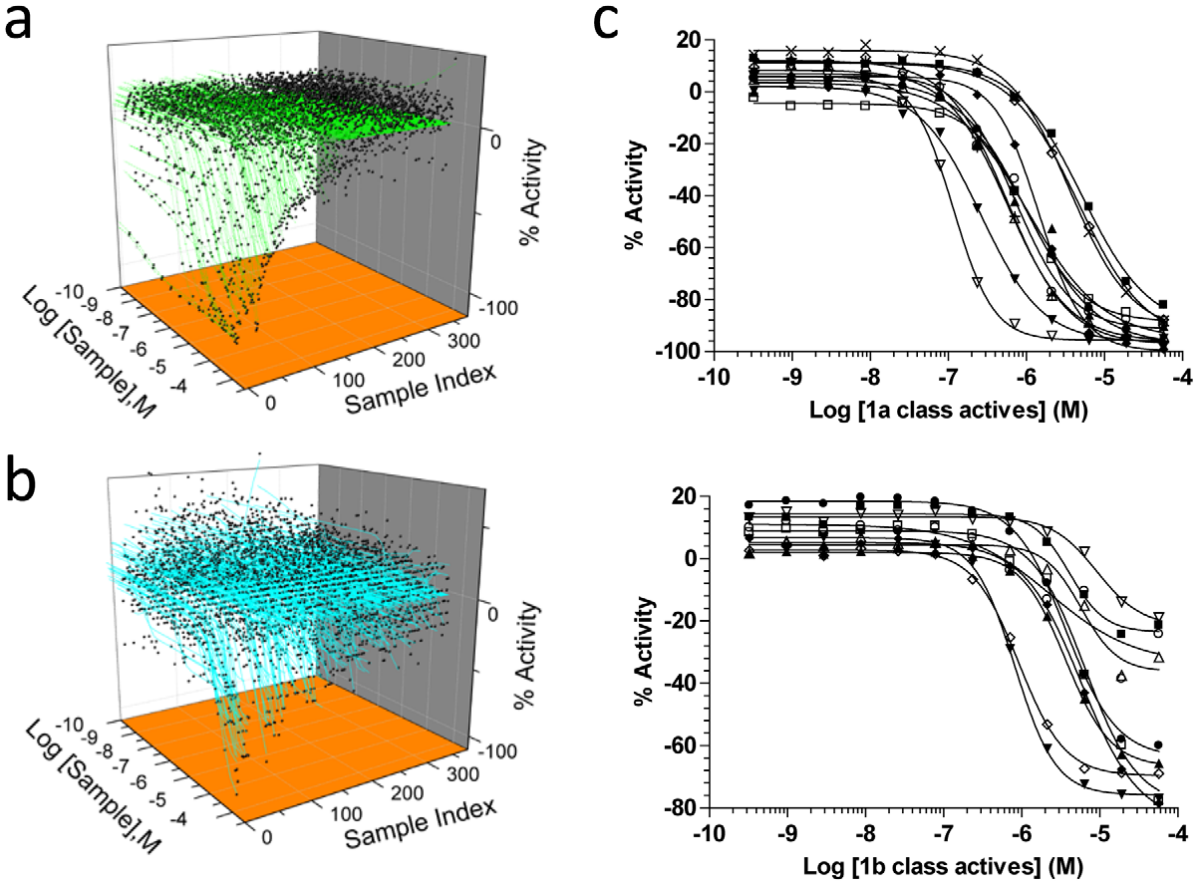
qHTS Profiling of GSK PKIS. (**a**) qHTS 3-axis plot of GSK PKIS activity against FLuc. (**b**) Same as **a.** except for RLuc. (**c**) Compounds in top panel display high quality curve fits (r^2^>0.9) fully titrating FLuc activity from 0% inhibition to 100% relative to a PTC124 control inhibitor. Those in the lower panel differ from the top group because the FLuc activity is not completely titrated compared to control (see Efficacy in **Table 2**). Compounds in curve class 1b typically display apparent incomplete titration of activity possibly due to not achieving a concentration for complete inhibition (∼10× IC_50_), or limiting solubility of the compound at the higher concentrations.

**Figure 2:**
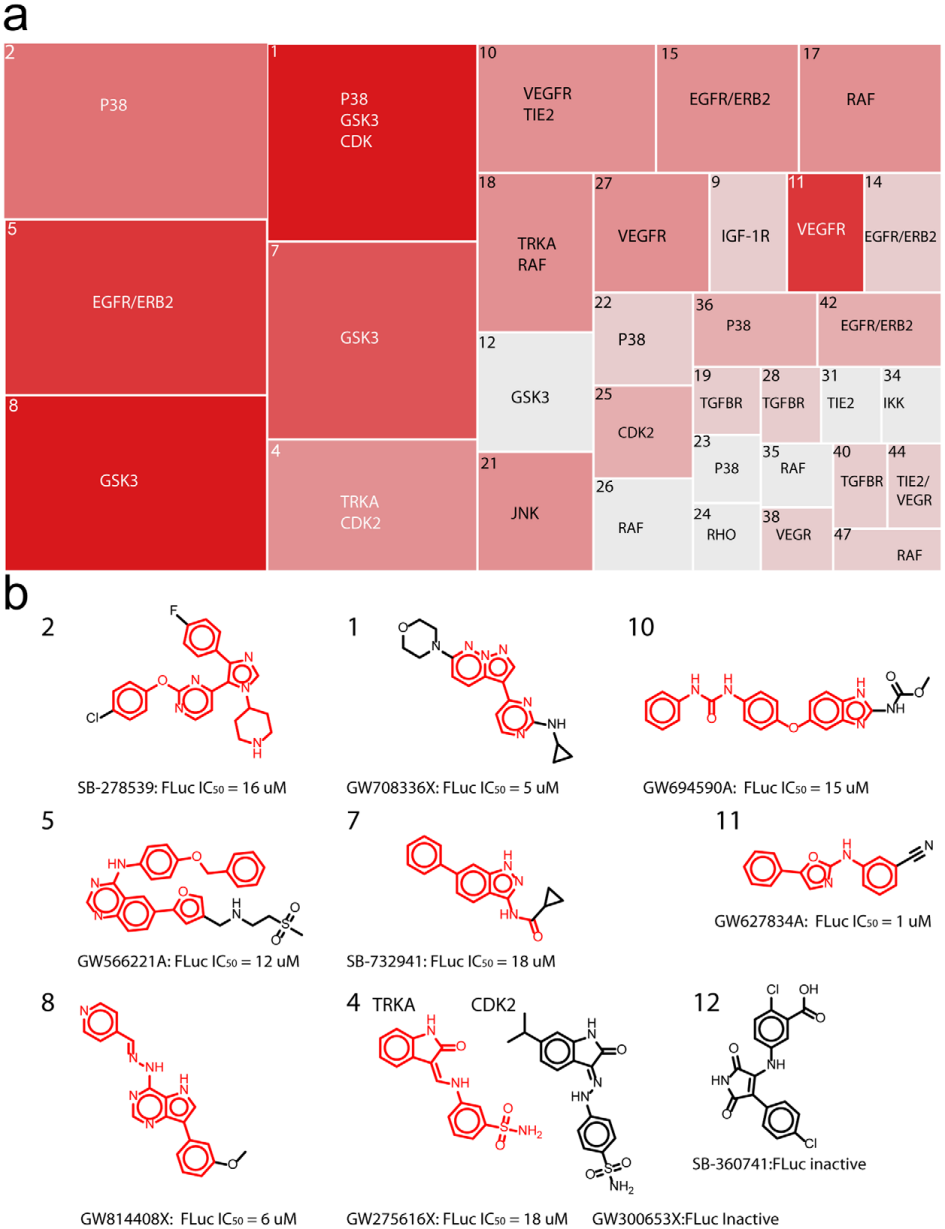
PKIS chemotype clusters with FLuc activity. (**a**) Treemap depicting 47 clusters based on Tanimoto similarity coefficients of 0.75. The size of each box represents the ratio of active to inactive compounds while the color represents the number of high quality (class 1 and 2) curves. The boxes, numbered by cluster are additionally annotated by the original target(s) associated with the cluster. Not shown are singletons or clusters where no FLuc inhibitors were identified. (**b**) Representative structures and FLuc potency values associated with prominent boxes on the treemap.

qHTS data was displayed after correction for plate-based aberrations and normalization using Origin software (OriginLab Corp., Northampton, MA) for the 3-axis plot of GSK PKIS activity against FLuc and RLuc (**Figure 1a, b**) or in Prism (GraphPad Software, San Diego, CA) as in **Figures 1c** and **2a, b**. The curve classification scheme for assignment of resulting curve-fit data into CRC classes was according to Inglese et al. [16] as follows: class 1 curves (far left) display two asymptotes, an inflection point, and r^2^≥0.9; subclasses 1a vs. 1b are differentiated by full (>80%) vs. partial (≤80%) response. Class 2 curves (second from left) display a single left-hand asymptote and inflection point; subclasses 2a (or 2.1) and 2b (2.2) are differentiated by a max response and r^2^, >80% and >0.9 or <80% and <0.9, respectively. Class 3 curves (second from right) have a single left-hand asymptote, no inflection point, and a response >3SD from the mean activity of the sample field. Class 4 (far right) defines those samples showing no activity across the concentration range.

### Clustering of FLuc actives

Clustering of active chemotypes was performed using SMILES to generate 2D structures and similarity was scored using the Tanimoto coefficient. Data was visualized in Spotfire (Spotfire TIBCO Software, Somerville, MA) where the hierarchy of similarity clusters can be visualized in a tree map (**Figure 2**).

## Results and Discussion

The availability of the 367-membered GSK PKIS will undoubtedly be a useful resource in the investigation of cellular networks and pathways, and also indicates a new collaborative spirit among pharmaceutical companies and public sector institutions [19], [20]. Numerous cellular process are under the control and regulation of protein kinases [21], and bioluminescent outputs, often based on luciferase reporter genes and sensors, are a prevalent means to monitor these activities [7]. In this study we have profiled the 367 compounds from the PKIS against FLuc and RLuc to derive the IC_50_ values of any compounds exhibiting an inhibitory effect on these enzymes (**Figure 1**). The concentration-response curves (CRCs) were categorized based on completeness of inhibition, presence of left and right asymptotes, and the quality of the curve-fit to the data (r^2^, **Figure 1**) [16]. We have found that approximately 6% of this library has significant FLuc inhibitory activity, with 18 compounds showing high quality CRCs and potencies <10 µM (**Table 2**), but far less activity (∼0.5%) against the non-homologous, ATP-independent luciferase from *Renilla reniformis.* Possible reasons for this difference include the fact that these kinase inhibitors bind in the nucleotide binding pocket of their respective kinase targets and such chemotypes may be accommodated by the FLuc ATP-binding pocket. This is in contrast to what has been observed for collections of luciferase inhibitors in which the primary mode of binding appears to be interaction with the luciferin pocket [11]. However, the extended binding cavity formed by the adjacent binding sites for ATP and D-luciferin in FLuc [10], [22] may be more accommodating to these kinase inhibitors.

**Table 2:** PKIS compounds with FLuc activity.

Sample ID	PubChem CID	Supplier ID	FLuc Parameters					RLuc Curve Class
			Curve Class	IC_50_ (µM)	Hill Coef	Efficacy	R^2^	
NCGC00241922-01	10276395	GW701427A	−1a	0.12	1.69	−101.6	0.999	−3
NCGC00242146-01	5329404	GW549390X	−1a	0.26	1.1	−98.6	0.998	4
NCGC00242171-01	5329831	GW809897X	−1a	0.58	1.11	−109.4	0.999	4
NCGC00242232-01	5329849	GW632046X	−1a	0.58	1.11	−104.4	0.998	4
NCGC00241923-01	25138030	GW759710A	−1a	0.73	1.21	−98.6	0.991	4
NCGC00242189-01	5329844	GW577921A	−1a	1.03	1	−101.8	1	4
NCGC00242006-01	16099859	GSK248233A	−1a	1.03	0.9	−98.9	0.988	4
NCGC00241897-01	5329845	GW627834A	−1a	1.03	1.21	−84.1	0.999	4
NCGC00241894-01	25138015	GW575808A	−1a	1.46	1.54	−105.8	0.998	4
NCGC00242047-01	10173796	GW779439X	−1a	3.66	1.1	−108.5	0.998	−2b
NCGC00242164-01	10193464	GW708336X	−1a	4.61	1	−107.5	0.999	4
NCGC00242070-01	53239963	GR105659X	−1a	5.17	1.01	−103	0.998	−2b
NCGC00242003-01	766948	GW572738X	−1b	0.82	1.66	−82.6	0.999	4
NCGC00242043-01	579429	SB-347804	−1b	0.92	1.48	−72.5	0.999	4
NCGC00242161-01	6539567	SB-711237	−1b	2.31	0.8	−44.8	0.901	4
NCGC00242024-01	53239959	GW578748X	−1b	3.66	1.33	−82.3	0.998	4
NCGC00241999-01	53239953	GW335962X	−1b	3.66	1.51	−68.8	0.999	4
NCGC00242120-01	53239969	GW284408X	−1b	4.11	1.93	−37.9	0.982	4
NCGC00242028-01	53239960	GW644007X	−1b	5.8	1.86	−40.7	0.972	4
NCGC00242020-01	5329852	GW575533A	−1b	5.8	1.17	−90	0.999	4
NCGC00242187-01	5329853	GW631581B	−1b	6.51	1.29	−88.5	0.995	4
NCGC00242107-01	44578563	GW806776X	−1b	9.19	1.51	−35.2	0.988	−2b

The chemotypes from the GSK PKIS that give rise to FLuc inhibition target both protein tyrosine kinases [23], [24], [25], [26], [27] and S/T kinases [28], [29], [30], [31], [32], [33], [34] as listed in **Table 3**. Structural clustering of the library members using Tanimoto coefficients [35] results in the 47 groups shown in **Figure 2A** and defined here by their original literature target kinase(s). Each cluster contains compounds showing a Taminoto coefficient of ≥0.75. The size of the box indicates a high ratio of FLuc inhibitory compounds relative to the total group size, based on quality curve class assignments (1a–3). The color of the box indicates the number of high quality curves represented in the cluster, with a darkly colored box (e.g., cluster 1, 5, 8 and 11) having a higher number of class 1 and 2 curves than lighter colored boxes. Among the representative chemotypes shown (**Figure 2B**) are several cores and linkers we have previously defined from the profiling of the large NIH Molecular Libraries collection which include the hydrazone linker of cluster 8 and the oxazole core of cluster 11 [11]. Additionally, this structure contains a pyrrolo[3,2-d]pyrimidine core which could suggest an ATP competitive chemotype. However a recent study describes a potent (IC_50_ values as low as ∼6 nM) series of pyrrolo[2,3-d]pyrimidine-based FLuc inhibitors that in fact bind to the luciferin pocket of firefly luciferase [36].

**Table 3:** Comparison of FLuc activity to PKI literature target activity.

GSK ID	FLuc IC_50_ (µM)	PKI Literature target	PKI IC_50_ (µM)	Literature cpd ID	Literature reference
GW549390X	0.26	VEGFR2	1.2	5	Harris, P.A., et al. 2005
GW632046X	0.58	VEGFR2	0.87	14	Harris, P.A., et al. 2005
GW577921A	1.03	VEGFR2	0.38	9	Harris, P.A., et al. 2005
GW627834A	1.03	VEGFR2	0.93	10	Harris, P.A., et al. 2005
GW575533A	5.8	VEGFR2	0.14	17	Harris, P.A., et al. 2005
GW631581B	6.51	VEGFR2	0.17	18	Harris, P.A., et al. 2005
GW572738X	0.82	JNK3	0.4	5e	Angell, R.M., et al. 2007
SB-347804	0.92	JNK3	3.16	5d	Angell, R.M., et al. 2007
SB-711237	2.31	GSK3α	>5	6	Witherington, J., et al. 2003
GW806776X	9.19	p38	0.075	3	Angell, R.M., et al. 2008
GR105659X	5.17	TRKA	0.006	5	Wood, E.R., et al. 2004
GW284408X	4.11	TRKA	0.007	7	Wood, E.R., et al. 2004
GW809897X	0.58	VEGFR	0.065	25	Sammond, D.M., et al. 2005
GSK248233A	1.03	RHO	0.002	6h	Stavenger, R. A., et al. 2007
GW701427A	0.12	VEGFR2	0.603	26	Hasegawa, M., et al. 2007
GW759710A	0.73	LCK	3.3	20	Bamborough, P., et al. 2007
GW575808A	1.46	LCK	0.19	5	Bamborough, P., et al. 2007
GW578748X	3.66	GSK3β	3.16	5	Peat, A. J., et al., 2004
GW644007X	5.8	GSK3β	2.51	6	Peat, A. J., et al., 2004
GW335962X	3.66	CDK2	0.36	19	Bramson, H. N., et al., 2001
GW779439X	3.66	CDK2/4	0.004/0.006	28	Stevens, K. L., et al. 2008
GW708336X	4.61	CDK	0.4	20	Stevens, K. L., et al. 2008

**Figure 3:**
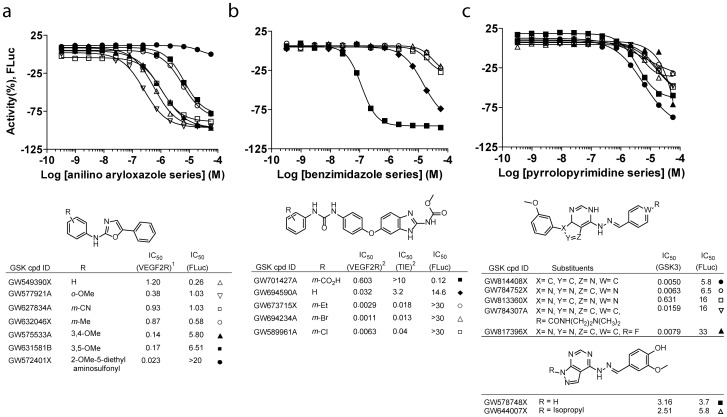
Representative PKIS series displaying FLuc inhibition. (**a**) Anilino-aryloxazole (Cluster 11, **Figure 2**), (**b**) benzimidazole-urea (Cluster 10, **Figure 2**), and pyrrolopyrimidines (Cluster 8, **Figure 2**) analogs displaying different FLuc inhibition SAR patterns. *Top,* CRC from this study for FLuc inhibition, *middle,* core scaffold for each series, *bottom,* activity of each analog against target kinase(s) and FLuc. Data for target kinases taken from references [24], [25], [30].

Three chemotypes emerge from this profiling study that illustrate how different structure activity relationships between FLuc and the target kinase inhibition can potentially confuse interpretation of the apparent protein kinase inhibitor activity in a cellular assay setting. The first chemotype belongs to an aryl-oxazole series developed in 2007 as inhibitors of VEGFR-2 tyrosine kinase [24]. For this aryl-oxazole series a clear SAR was observed for FLuc inhibition that inversely tracked with the VEGFR-2 activity. The most potent VEGFR-2 PKI, GW572401X (23 nM) displayed the least FLuc activity (20 uM), while the most potent FLuc inhibitors were among the lower activity VEGFR2 series analogs (**Figure 3A**). The implications of this would primarily revolve around the use of one of the low potency or ‘inactive’ analogs as a negative control. Such controls are important in order to ascertain that the activity of the active compound derives from inhibition of its targeted kinase and is not an off-target property of the series [37]. However, if for example GW549390X is used as a negative/low potency control for GW572401X in an FLuc reporter assay the effect of GW549390X on FLuc activity could confound the comparison to the observed activity of GW572401X. Further, in an unbiased HTS this series would potentially lead to puzzling results. For example, the ability of even low potency FLuc inhibitors to stabilize FLuc and increase its cellular half-life [11] would increase measured reporter activity compared to non-liganded, non-stabilized FLuc.

The second chemotype series was developed as potent inhibitors of TIE-2 and VEGFR-2 tyrosine kinase [25]. Interestingly, most members of this benzimidazole-urea series (**Figure 3B**) have modest to no FLuc activity, however one analog, GW701427A displays unusually high FLuc inhibition (120 nM). GW701427A contains a meta-carboxylic acid on the aryl ring position that highly influences binding to VEGFR-2/TIE-2. Our previous work has shown that appropriately placed aryl carboxylates on FLuc ligands can result in ATP-dependent FLuc-mediated adenylation of the ligand [22]. Such adenylated ligands can greatly stabilize the FLuc reporter over the time course for the assay, and with facilitated release during the detection phase of the assay [22], results in an apparent increase in reporter activity [11].

A third chemotype originally designed as a GSK3 inhibitor displays weak but consistent FLuc inhibition for a number of cluster 8 members (**Figure 2A**). These contain a hydrazone linker attaching a pyrazolopyrimidine core to an aryl moiety (**Figure 3C**). We have observed that this hydrazone linker is enriched in FLuc inhibitors (**Figure S1**), and in particular those compounds in which the attached aryl group is a meta-substituted benzoic acid can be quite potent FLuc inhibitors [11].

Because the EC_50_ for reporter stabilization is mirrored by the IC_50_ for reporter enzymatic activity inhibition even by relatively weak inhibitors of FLuc or RLuc (IC_50_ between 5 and 10 µM) one can observe appreciable stabilization of reporter half-life at typical screening concentrations [11]. For kinase-regulated pathways, networks or cellular processes, an apparent reporter activation resulting from treatment with a kinase inhibitor chemotype could be misinterpreted as affecting a kinase that represses the pathway activity, for example as in GSK3β-mediated transcription factor (e.g., β-Catenin) phosphorylation leading to ubiquitination, nuclear exit, and decreased nuclear transcription [38].

**Figure 4:**
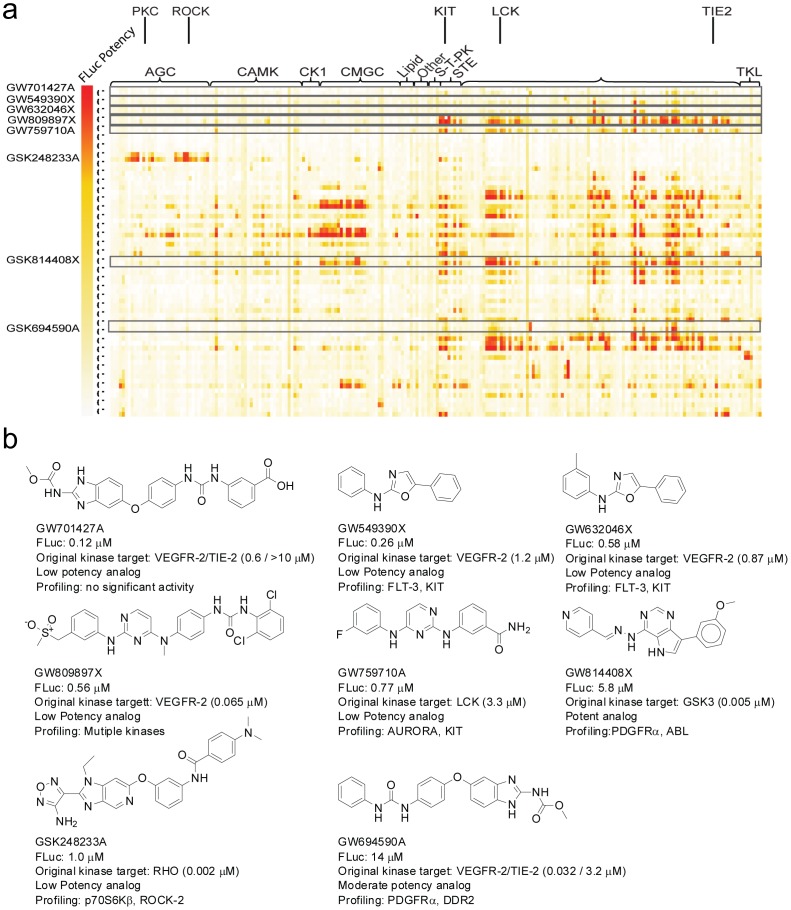
Kinome profiling relating FLuc inhibitory activity vs PKI activity. (**a**) Heatmap depicting the activity of the 367 member GSK PKIS against 224 protein kinases, assayed at two concentrations (0.1 and 1 µM). The vertical heatmap bar depicts the potency of these compounds (IC_50_) against FLuc. (**b**) Several compounds representative of those displaying FLuc activity between <1–14 µM IC_50_ values are shown and highlighted on the heatmap above. Profiling: kinases showing >80% inhibition at 100 nM are named.

Cross-examination of FLuc inhibitory PKIS chemotypes against kinome activity as derived from GSK PKIS profiling data available in Chembl [39] (https://www.ebi.ac.uk/chembl/) is shown in **Figure 4**. The PKIS library was rich in tyrosine kinase, CK1, and CMGC family inhibitors with less activity at other families. Of note, many of the potent FLuc inhibitors were found to be associated with broad activity in the tyrosine kinase family, with only one of these compounds (GSK248233A, **Figure 4**) showing activity in the AGC family. Two related analogs, showing activity at FLT-3 and KIT, were also found to be highly potent inhibitors of FLuc. Phenyl-oxazoles have been noted previously as FLuc inhibitors [11], with many of these FLuc inhibitors showing competition with D-luciferin. In this kinase inhibitor series, the aniline side-chain present in GW549390X and GW632046X could allow binding to the ATP pocket of FLuc. Interestingly, the most potent FLuc inhibitor in our panel showed little activity at any of the protein kinases, a result consistent with divergent SAR for higher potency FLuc inhibitors.

Given the paradoxical observation that inhibitors of FLuc and RLuc can prolong the cellular half-lives of these enzymes [13], [40], thus confounding the interpretation of experimental results obtained with such compounds, the present data set should be useful in helping to consider if the potential exists for observing apparent pathway activation resulting from reporter stabilization. We have noted this phenomenon in several cell-based assays [11], [13], [40], [41], [42], [43]. Further, the effect is observed for a range of IC_50_ values from nM to µM. Because the scaffolds associated with FLuc inhibition, for example, are present in many chemotypes including some FDA-approved drugs (see PubChem AID 624030) [43] the effect of FLuc stabilization or direct inhibition can be found among well-characterized compounds as well as general chemical screening libraries. This point also illustrates that drug libraries, or highly annotated mechanism of action libraries, are just as susceptible to superficial activity due to reporter interference. Therefore the possibility of direct reporter activity needs to be understood before generating a target hypothesis from the data, otherwise the resulting pathway maps derived from luciferase reporter gene assays will be biased by luciferase activity. Overall this study points to the need for orthogonal reporter systems if reporter gene assays are used to characterize compounds. For example, both GW549390X and GW632046X are potent FLT-3/KIT protein kinase inhibitors but also show potent FLuc inhibitory activity. Likewise the PKA inhibitor H89 is also a RLuc inhibitor [12]. Therefore, to distinguish pathway modulation by protein kinase inhibition versus reporter inhibition one will ideally understand the inhibition profile of the enzyme reporter (as we performed here for the GSK PKIS) and/or employ a reporter known to be refractory to the compounds of interest. The use of paired cell lines each expressing an unrelated reporter such as FLuc/RLuc [40] or FLuc/β-lactamase [43] represents an effective strategy to rapidly identify reporter–biased effects from desired biological activity. Recently we have developed a coincidence biocircuit based on this concept that allows expression of two non-homologous reporters from the same transcript in a stable stoichiometry providing a facile system for construction of orthogonal reporter systems in a single cell line [44].

Consistent with previous observations from broad screening efforts [11], FLuc inhibitors were identified from a variety of structural motifs. Visual comparison with known FLuc inhibitors, for example the aminooxazole series exemplified by GW577921X and the control compound PTC124, may help to identify FLuc inhibitors and rationalize the activity with this in mind. On the other hand, structures such as GSK248233A appear distinct from known FLuc inhibitors, underscoring the need for verification of FLuc activity when employing this technology in cellular screening.

## Supporting Information

Figure S1 **Enrichment analysis for hydrazone linker in FLuc inhibitors.** (**a**) The hydrazone linker used to query the MLPCN library and FLuc actives list. (**b**) Frequency of the linker in the MLPCN (n = 364,105) vs (**c**) frequency of the linker appearing in FLuc actives (n = 12,353) of curve classes 1a–3.
(EPS)
